# ZNF217-USP15 signaling loop regulates oncogenic phenotypes in ovarian cancer cells

**DOI:** 10.64898/2026.08.30.748158

**Published:** 2026-08-31

**Authors:** Ayokunnumi Ogunsanya, Fatimah Alfaran, Swethakumar Basavarajaiah, Achuth Padmanabhan

## Abstract

ZNF217 is an established oncogenic transcription factor that promotes cancer progression and therapeutic resistance; however, the mechanisms regulating ZNF217 protein abundance remain poorly understood. Here, we identify ubiquitin-specific peptidase 15 (USP15) as a critical regulator of ZNF217 stability and define a reciprocal USP15–ZNF217 signaling loop that sustains malignant phenotypes in ovarian cancer. Stable overexpression of ZNF217 in OVCA420 ovarian cancer cells enhanced proliferation, epithelial–mesenchymal transition, migration, invasion, and extracellular matrix adhesion. Notably, ZNF217 overexpression increased USP15 protein abundance without altering USP15 mRNA levels, whereas ZNF217 depletion reduced USP15 protein levels, suggesting post-transcriptional regulation. Conversely, USP15 depletion markedly reduced ZNF217 protein abundance while increasing ZNF217 mRNA levels, indicating that USP15 regulates ZNF217 predominantly at the post-transcriptional level. Proteasome inhibition restored ZNF217 protein levels following USP15 depletion, further demonstrating that USP15 promotes ZNF217 protein stability. Functionally, USP15 depletion in ZNF217-overexpressing ovarian cancer cells suppressed proliferation and multiple metastatic phenotypes, including migration, invasion, extracellular matrix adhesion, anoikis resistance, and multicellular aggregate formation. In vivo, USP15 depletion significantly reduced tumor progression and metastatic burden and prolonged survival in mice bearing ZNF217-driven ovarian tumors. Furthermore, USP15 depletion enhanced the sensitivity of ZNF217-overexpressing cells to carboplatin, paclitaxel, and doxorubicin. Collectively, these findings identify USP15 as an upstream regulator of ZNF217 protein stability and reveal a positive-feedback loop between USP15 and ZNF217 that reinforces oncogenic signaling. Targeting USP15 may therefore represent an indirect therapeutic strategy for suppressing ZNF217-driven ovarian cancer, particularly given the challenges associated with directly targeting oncogenic transcription factors.

## Full Text Availability

The license terms selected by the author(s) for this preprint version do not permit archiving in PMC. The full text is available from the preprint server.

